# β-Defensins in the Fight against *Helicobacter pylori*

**DOI:** 10.3390/molecules22030424

**Published:** 2017-03-07

**Authors:** Raffaela Pero, Lorena Coretti, Ersilia Nigro, Francesca Lembo, Sonia Laneri, Barbara Lombardo, Aurora Daniele, Olga Scudiero

**Affiliations:** 1 Dipartimento di Medicina Molecolare e Biotecnologie Mediche, Università degli Studi di Napoli “Federico II’, 80131 Napoli, Italy; lorena.coretti@tiscali.it (L.C.); barbara.lombardo@unina.it (B.L.); 2 CEINGE-Biotecnologie Avanzate Scarl, Via G. Salvatore 486, 80145 Napoli, Italy; ersilia.nigro@unina.it (E.N.); aurora.daniele@unina2.it (A.D.); 3 Dipartimento di Scienze e Tecnologie Ambientali Biologiche Farmaceutiche, Seconda Università degli Studi di Napoli, Via G. Vivaldi 42, 81100 Caserta, Italy; 4 Dipartimento di Farmacia, Università degli Studi di Napoli ‘Federico II’, Via Montesano 49, 80131 Napoli, Italy; frlembo@unina.it (F.L.); sonia.laneri@unina.it (S.L.)

**Keywords:** defensins, *Helicobacter pylori*, gastric disease, antimicrobial therapy

## Abstract

Antimicrobial peptides (AMPs) play a pivotal role in the innate immune responses to *Helicobacter pylori (Hp)* in humans. β-Defensins, a class of cationic arginine-rich AMPs, are small peptides secreted by immune cells and epithelial cells that exert antimicrobial activity against a broad spectrum of microorganisms, including Gram-positive and Gram-negative bacteria and fungi. During *Hp* infections, AMP expression is able to eradicate the bacteria, thereby preventing *Hp* infections in gastrointestinal tract. It is likely that gastric β-defensins expression is increased during Hp infection. The aim of this review is to focus on increased knowledge of the role of β-defensins in response to *Hp* infection. We also briefly discuss the potential use of AMPs, either alone or in combination with conventional antibiotics, for the treatment of *Hp* infection.

## 1. Introduction

*Helicobacter pylori (Hp)* is a Gram-negative, spiral-shaped flagellate bacterium involved in several gastric diseases [1]. The most common and serious complications of *Hp* infection include peptic ulcer disease (10%), gastric adenocarcinoma (1%–3%), and primary gastric mucosa-associated lymphoid tissue (MALT) lymphoma [2]. While the role of *Hp* in the pathogenesis of peptic ulcer disease and gastric cancer has been clarified, contradictory results have emerged concerning its correlation with morbid obesity [3]. Approximately, half of the world’s population is infected with *Hp* although the prevalence differs between countries and depends on various factors such as *Hp* virulence factors, inflammatory responses, or enviromental influences, which finally influence the host-pathogen interactions [4]. Human β-defensins (HBD) play a pivotal role in the immune response of the gastrointestinal epithelium to *Hp*-infection [5]. In this review we aimed to focus on what is currently known about interaction of *Hp* and host focusing on biological functions of β-defensins. Moreover, we briefly discuss the potential use of AMPs (Antimicrobial peptides) for the treatment of *Hp* infection.

## 2. HP Virulence Factors

Two well characterized *Hp* virulence factors are the cytotoxin-associated A (CagA) and vacuolating cytotoxin A (VacA) proteins. The CagA gene is a part of a 40 kb cluster of genes (Cag Pathogenicity Island, *cag*PAI), some of which are more frequently associated with severe gastric inflammation, ulceration and an increased risk of gastric cancer [6].

CagA is introduced into cells through the T4SS (type IV secretion system) together with peptidoglycans. In the host, intracellular peptidoglycan interacts with the recognition molecule NOD1 (nucleotide-binding oligomerization domain), which acts as a sensor for peptidoglycan components originating from Gram-negative bacteria. This interaction leads to activation of Nuclear Factor Kappa B (NF-κB), allowing the secretion of pro-inflammatory cytokines and chemokines.

The *cag*PAI may be present fully, partially, or absent. Strains with a functional *cag* T4SS are strongly associated with increased gastric cancer risk compared to strains lacking CagA which may also induce inflammation via other *cag*PAI-dependent mechanisms [7].

VacA gene encodes an 87 kD protein that induces vacuolation of epithelial cells [8]. The vacA gene is present in all strains of *Hp* but is highly polymorphic. In fact, vacA presents two alternative allelic variants parts for the *s* region, encoding the signal peptide (*s1* or *s2* allele), intermediate (*i1*/*i2*) and mid- (*m1*/*m2)* [9]. The mosaic combination of *s* and *m* region allelic types determines the production of the cytotoxin and is associated with pathogenicity of the bacteria [10]. For example, the vacA *s1* and *i1* alleles are associated with increased risk of peptic ulceration, atrophy and gastric adenocarcinoma [11].

The neutrophil-activating protein (NAP) of *Hp* represents another important factor involved in eliciting the host Th1 response by stimulating pro-inflammatory cytokines and chemokines secretion from neutrophils and monocytes [12].

Moreover*, Hp* proteins HP0175 and HP0986 directly interact with host tissues via TLR4 and Tumor necrosis factor receptor-1, respectively [13,14]. These virulence factors are also involved in deregulation of various oncogenic pathways, such as p53, wnt/β-catenin, NF-κB and PI3K/Akt pathways [15].

In the field of inflammatory gastric diseases the research is mainly focused on dysregulation of the adaptive immune responses, including a primary disturbed barrier function of the gastric tract and the system of innate immunity [16].

As a mucosal surface, the gastric tract is protected by an innate antimicrobial system consisting of numerous peptides. Playing an important role in host innate defense these peptides indirectly confer epithelial barrier function as an adjunct to specific immunity [17].

Previous studies have reported the induction of several potent antimicrobial cationic defensin peptides during the early stages of infection with various bacterial species [18,19,20]. These peptides are constitutively expressed or induced and contribute to innate and adaptive immunity through effector and regulatory functions [21]. Since defensins are effective against Gram negative and positive bacteria, fungi, parasites (*Cryptosporidium Parvum*, *Toxoplasma Gondii*, *Trypanosoma cruzi*, and viruses, they are important components of first-line host defenses [22,23,24]. The increasing knowledge about antimicrobial peptides (AMPs) and their role in innate immunity has added important new aspects to the current understanding of the pathogenesis of inflammatory gastric cancer [25].

### 2.1. Antimicrobial Peptides

AMPs are a large group of effector molecules of the innate immune system with a broad spectrum of biological activities. AMPs are highly conserved components of the innate immune response, well known since 1922 when they were first isolated from human tears by Fleming and Ridley [26,27]. AMPs provide protection against environmental pathogens, acting against a large number of microorganisms, including bacteria, fungi, yeast and virus. Besides their antimicrobial function, they exert multiple roles as mediators of inflammation with impact on epithelial and inflammatory cells influencing diverse processes such as cell proliferation, immune induction, wound healing, cytokine release, chemotaxis and protease-antiprotease balance. Their production is either constitutive or induced, depending on organisms, cell type and peptides. The majority of these small peptides (fewer than 100 amino acids) share a common feature: the presence of a positive net charge of +2 to +9 due to an excess of positively amino acids (i.e., arginine and lysine). The mechanism of action is based on their net positive charge. AMPs interact electrostatically with negative charges of microbial cell membranes (i.e., phospholipids), thereby increasing the membrane permeability and finally resulting in cell death [28,29].

AMPs may be organized into three basic categories concerning target: (i) plasma membrane–active peptides are thought to act in a multistage process where they electrostatically bind to a membrane surface, aggregate to form superstructures, and disrupt membrane integrity; (ii) a second group of peptides act on intracellular targets to inhibit transcriptional, translational or other processes; (iii) and cell wall–active peptides target precursors, mechanisms, and/or essential intermediates in peptidoglycan, lipopolysaccharide (LPS) or other biosynthetic pathways interfering with functional cell wall synthesis and ensuing bacterial replication.

To date, over 2500 peptides have been identified and recorded in the database of antimicrobial peptide [30]; usually, AMPs are classified based on secondary structural features, such as cathelicidins (linear α-helical peptides), defensins (β-strand peptides connected by disulfide bonds), and bactenecins (loop peptides). In humans, two main categories of antimicrobial peptide play a key role: cathelicidins and defensins, both secreted by epithelial cells of many tissues as respiratory, urogenital and gastro-intestinal tracts [31].

### 2.2. Defensins: The Classification

Defensins, a class of cationic AMPs arginine rich, are small peptides highly conserved during the evolution from plants to humans [32]. They are secreted by immune cells as neutrophils, monocytes and epithelial cells of gastro-intestinal, pulmonary, urogenital epithelia, skin and placenta [33,34]. They exert antimicrobial activity against a broad spectrum of microorganisms, Gram-positive, -negative, fungi and viruses [35] and exert chemotaxis toward dendritic cells, monocytes and lymphocytes. Three defensin subfamilies have been deeply described: α-, β- and θ-defensins. Each subfamily has a conserved motif that includes six cysteine residues that form three intramolecular disulfide bonds with a characteristic and different pattern of pairing. These peptides are produced as pre-pro-peptides of 94–95 amino acids that undergo two cleavage processes releasing the mature peptides of 29–34 amino acids [32]. The mature defensins of the three subfamilies share some common features: (i) short sequence: 18–50 amino acids (5–7 KDa); (ii) net cationic charge; (iii) presence of six cysteine residues; (iv) three intramolecular disulphide bonds due to the presence of six cysteines; absence of post-traductional modifications. Otherwise, they differ for: (i) the length of peptide segment between the six cysteines; (ii) the pairing of the cysteines that are connected by disulfide bonds; (iii) the tridimensional conformation.

In humans, α- and β-defensins are produced by two distinct genes evolved from a common ancestral gene; this process probably is due to the evolutionary response of the immune system to the continuous ecological and environmental changes [36]. Both α- and β-defensin genes, organized into distinct clusters, have been mapped to band 8p23.1. The β-defensin gene family consists of 40 family members at five gene loci in humans and more than 50 genes on chromosomes 8, 4, 6, 11, and 20. The main cluster is on chromosome 8. In human, genes on the chromosome 8 have a highly copy number variation (CNV), varying between 2 and 7 copies per genome.

No human θ-defensins have been isolated to date, but humans have three θ-defensin pseudogenes that contain premature stop codons. In non-human primates, θ-defensins have been isolated from neutrophils and from bone marrow. The θ-defensins are structurally different from α- and β-defensins: they are in fact cyclic peptides. Moreover, the gene encoding for θ-defensins originates from the mutated gene of α-defensins [37]. The precursor of the θ-defensins is a shorter paralogous version of the α-defensins because it is truncated by the insertion of a stop codon. The resulting mature peptide of 18 amino acids presents the classical six cysteines and three disulfide bridges that, making the structure very rigid, give raise the typical cyclic structure.

### 2.3. Structure and Function: Human α-Defensins

Human α-defensins are arginine-rich peptides, containing 29–35 amino acids. Humans have six α-defensins termed human neutrophil proteins 1 through 4 (HNP1-4) and human defensins 5 and 6 (HD5- and 6). HNP1-4 are secreted by granulocytes while HD5- and 6 are produced by Paneth cells, in the crypts of the small intestine. α-Defensins have been isolated from neutrophils and, more specifically, within the azurophilic granules in which HPN1-3 represent about the 50% of protein content while HNP4 is present at low concentrations [37]. Defensins HNP1- and 3 are also present in B and natural killer lymphocytes. Defensins HD5- and 6 are defined as enteric defensins because they have been found in Paneth cells of the small intestine and in the epithelial cells of the female urogenital tract (endometrium, and fallopian tubes) [38]. From a functional point of view, α-defensins play a key role in oxygen-dependent destruction of phagocytised microorganisms.

They are synthesized as pre-pro-peptides (93–100 aminoacids) with a 19-amino acid signal peptide and a 41–51 amino acid anionic pro-segment. The core of α-defensin molecules consists of three β strands that connect cysteines 1–6, 2–4 and 3–5. In addition, in α-defensin, but not in the β class, the β sheet is flanked by an α helical segment of variable length, corresponding to the N-terminal domain. This particular conformation and charges distribution are probably responsible for the antimicrobial activity of defensins [33].

### 2.4. Human β-Defensins

Human β-defensins (HBDs) are peptides of about 35 amino acid residues, including six cysteine residues that create three disulfide bonds (1–5, 2–4, 30–6). They are expressed predominantly in epithelial tissues, which provide the first line of defense between human and the environment. HBDs possess two main activities: antimicrobial and chemotactic.

So far more than 50 HBD genes have been found in the human genome. In the last years, a number of β-defensins have been studied, but the best characterized are HBD1, HBD2, HBD3 and HBD4 [39].

HBDs manifest antimicrobial activity against several microbes, including Gram-positive and Gram-negative bacteria, fungi and viruses [40,41]. In vitro assays demonstrate broad-spectrum activity of all β-defensins against Gram-positive and -negative bacteria, viruses (predominantly enveloped), and fungi, with minimal inhibitory concentrations in the μg·mL^−1^ range. However, different HBDs are selective in their activity. HBD1 is more active against Gram-negative bacteria, while HBD-2, HBD-3 and HBD-4 are also active against Gram-positive bacteria and yeast [40,42]. Furthermore, HBD1 and HBD2 are sensitive to salt, whereas HBD3 is the only salt-resistant β-defensin. The salt resistance is a very important aspect in patients affected by cystic fibrosis: several studies have demonstrated that the system of β-defensins in the airway epithelium of these patients is completely inactivated by high salt concentrations [43]. HBD1 was firstly isolated from a patient undergoing hemofiltration dialysis; later it has been later found in a large variety of tissues [44,45]. hBD1 is expressed in that epithelia directly exposed to the external environment such as lung, mammary glands, salivary glands, kidney, pancreas and prostate [40,46]. HBD1 is the only defensin that can be expressed both in a constitutive or inducible manner consequent to exposure to bacteria or pro-inflammatory cytokines (as interleukin-1β (IL-1β), tumour necrosis factor-α (TNF-α) and interferon-γ (IFN-γ) [47]. A second family member, HBD2, was firstly isolated from psoriatic scale extracts; successively it was found in lung, stomach, urogenital system and intestine [48,49]. HBD2 is not expressed at basal level but it is inducible after exposition to bacteria, lipopolysaccharide and pro-inflammatory cytokines as TNF-α and IL-1β [47]. HBD3 was initially isolated from epidermal keratinocytes from patients with psoriasis. It is induced by IFN-γ, TNF-α and bacteria in lung and intestine [50]. Finally, HBD4 was first isolated in lung tissues. It is not expressed at a basal level but can be up-regulated by Gram-negative and Gram-positive bacteria and by IFN-γ and TNF-α [51]. HBD3 and hBD4 are expressed also in the endometrium [52].

Thanks to their chemotactic activity, β-defensins have been described as molecules that provide a link between the innate and adaptive immune responses. Chemoattractant activity toward dendritic cells, memory T cells and mast cells has been reported for hBD2 and hBD3 [40] and both HBD3 and HBD4 are chemotactic toward monocytes [50,53]. The chemotactic activity of HBD-s towards immune cells seems to be mediated by the binding of defensins to the chemokine receptors CCR6, CXCR2 and CXCR4 [32,54]. Recently, we used a functional proteomic approach to search for cell surface receptors that could play a role in the interaction and internalization of HBD3 in A549 cells. CD98, a type II transmembrane protein involved in amino acid transport, cell adhesion, inflammation, immune response and attachment of enteric bacterial pathogens was identified as a novel receptor for HBD3 [55].

### 2.5. Killing Mechanism: Bacteria

Although the killing mechanism of defensins is not yet clear, it seems that the permeabilization of target membranes is the crucial step in defensin-mediated antimicrobial activity. In fact, it has been shown that bacteria treated with β-defensins can be permeabilized by small molecules. Moreover, the conditions which interfered with permeabilization of bacterial membranes prevent the loss of viability of bacteria, indicating permeabilization mechanism for microbial killing [56]. Further, in experiments with artificial membranes characterized by the presence of negative charges, defensins formed channels [57]. All these observations are consistent with the idea that the insertion of defensin molecules into the membranes depends on electrostatic forces [58]. Moreover, the activity of defensins against artificial membranes was diminished in the presence of increased salt concentrations, supporting the importance of electrostatic forces between the anionic phospholipid headgroups and the cationic defensins [57].

Considering these data, the most reliable mechanism of antimicrobial activity is the following: the positive residues of HBD-s interact with negative charges of plasma membranes of microorganisms through an electrostatic recognition. Successively, with the amphipathic portion, defensins penetrate into the lipid bilayer inducing the permeabilization of membranes. This appears to be the lethal event [59,60].

However, stable pore formation is not the only mechanism of defensin pathogen killing. Probably, HBDs are able to inhibit RNA, DNA and protein synthesis and induce the synthesis in epithelial cells of cytokines, as INF (interferon γ), which contributes to bacteria and viruses elimination. It is also possible that several mechanisms cooperate to induce pathogen death [61].

#### 3. β-Defensins and *Hp* Infection

In *Hp-*infected patients and/or infected human gastric epithelial cells in vitro, elevated levels of HBD2, HBD3, and HBD4 have been shown in the gastric mucosa [62,63,64,65,66]. As a consequence of *Hp* interactions with the epithelium, pro-inflammatory chemokines and cytokines, including IL-8, IL-1, tumor necrosis factor α (TNF), IL-6, IL-12, CCL2-5, CCL20, and CXCL1-3, are up-regulated in the infected gastric mucosa. Chemokines lead to the recruitment of neutrophils that contribute to gastritis by secreting inflammatory cytokines and releasing tissue damaging factors. Neutrophils also phagocytose bacteria, and within the phagolysosomes the bacteria are exposed to bactericidal factors, including myeloperoxidase and matrix metalloproteinases, which degrade cell walls and proteins, and ROS and RNS, which induce DNA damage [67,68].

Recent studies emphasize that bacteria can subvert host immune responses not only by direct recruitment of host inhibitory receptors [69,70] but also through virulence factors that resemble intermediates of host inhibitory signaling and interfere with defense functions [71,72,73].

The most common inhibitory motif is the immunoreceptor tyrosine-based inhibitory motif (ITIM). Recruitment of ITIM receptors results in ITIM tyrosine phosphorylation and recruitment of downstream mediators containing Src homology 2 (SH2) domains, such as SHP-1, SHP-2, SHIP, and Csk. Next, dephosphorylating signaling intermediates cause them to act on their respective targets to dampen inflammatory signals relayed by activating receptors [74].

**Figure 1:**
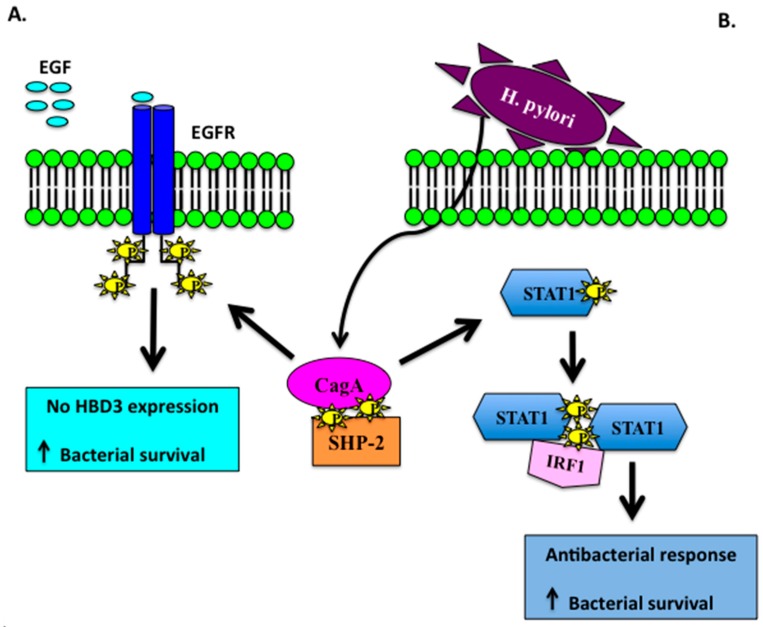
Schematic representation of *Hp* infection in gastric epithelium. (**A**) CagA induces EGFR dephosphorylation abrogating HBD3 expression and increasing bacterial viability; (**B**) Cag A inhibits IFN-γ-dependent STAT1 signaling which in turn promotes bacterial survival.

CagA represents the first identified bacterially encoded effector containing tyrosine-based motifs resembling ITIMs. During *Hp* infection, a type IV secretion system exports CagA into host cells. Translocated CagA undergoes tyrosine phosphorylation in the host cells and directly mediates SHP-2 (Src homology domai P-2) activation by binding to SH2 domains in a phosphorylation-dependent manner [75]. Following activation by CagA, SHP-2 dephosphorylates the intracellular domains of EGFR, thereby abrogating HBD3 synthesis (mRNA and protein) and increasing bacterial viability [76]. Morover, CagA is also enable to modulate epithelial cell inflammatory responses by preventing the induction of IFN-γ-dependent STAT1 phosphorylation and IRF1 transactivation in targeted epithelial cells (Figure 1). Such inhibition of a key signal transduction cascade could represent bacterial adaptation for modulating the host mucosal immune response to promote bacterial survival in the stomach [73].

It has previously shown both in vitro and in vivo increased mRNA expression of gastric β-defensins during *Hp* infection [5,77,78,79]. Morover, HBD2 gene expression required the presence of *cag*PAI and is NOD1-dependent. In contrast, HBD3 expression is NOD1-independent but relied on epidermal growth factor receptor (EGFR)-mediated ERK activation, suggesting distinct temporal functions for the two antimicrobial peptides during infection [64]. NOD1 signalling pathway results in NF-κB translocation to the nucleus, but NOD1 also activates the transcription factor AP1 via ERK- and p38-dependent pathways [7,80].

A direct consequence of NOD1 signalling is efficient killing of *Hp* by HBD2, in NOD1-activated gastric epithelial cells [81]. The idea that *Hp*-induced activation of NF-κB depends on NOD1 has recently been challenged by a report showing that the introduction of siRNA (small interfering RNA) specific for NOD1 does not alter the nuclear translocation of the NF-κB subunit p65 [82]. This new study provides evidence for an alternative NOD1-dependent signalling pathway, which activates the IRF3 and IRF7 transcription factors to induce the production of type I IFNs that are required for *Hp*-specific cytokine and chemokine responses, and infection control.

### 3.1. Hp-Induced Gastritis

The antimicrobial response by host epithelial cells plays a crucial role in bacterial adherence to the epithelium and in the development of *Hp*-induced gastritis. Chronic *Hp* infection leads to local inflammation of the gastric mucosa (gastritis). Disease risk increases with the level of inflammation, but the pattern of inflammation determines the disease outcome. Host genetic factors, bacterial virulence, environmental factors, and age of infection, influence the distribution of resulting gastritis [83].

Chronic *Hp*-gastritis involves the incisura angularis and subsequently the antrum and less frequently the corpus. *Hp* infection with strains that contains the *cag*PAI secrete a number of bacterial products that cause a severe injury of the gastric epithelium [84,85,86].

*Hp* infection leads to a significant induction of HBD-2 while the defensin gene expression caused by non-*Helicobacter* gastritis is much less pronounced [87]. *Hp* induces gastric epithelial cells to upregulate the endogenous production of HBD2 and this upregulation is mediated by the cytosolic pattern recognition receptor NOD1 (nucleotide- binding oligomerization domain 1) [81].

A single nucleotide polymorphisms (SNP G-52) in the *DEFB1 gene* correlated patients with chronic active *Hp*-induced gastritis, suggesting an involvement of the constitutive expressed HBD-1 in susceptibility to this form of gastritis [88].

Bajaj-Elliott et al., showed for the first time that in vitro mRNA expression of HBD1 is not only constitutive but can be further modulated during infection. This study confirmed marked upregulation of HBD2 by *Hp* and evaluated expression of these genes in *Hp* induced gastritis tissue, observing a significant increase in HBD1 and HBD2 mRNA in gastritis compared with control. Immunohistochemical analyses revealed a parallel increase in both HBD1 and HBD2 peptide expression during bacterial induced inflammation. The study suggests that surface expression of HBD1 and HBD2 may constitute an important component of the host innate defence to potential harmful stimuli in the human stomach [5].

Hamanaka et al., revealed that the gastric tissue of patients with *Hp* infected gastritis overexpressed HBD2 mRNA, as assessed by RT-PCR, while those from *Hp* negative patients showed only faint hBD2 expression, suggesting that *Hp* colonisation induces hBD2 expression in the gastric mucosa. They also found a considerable difference between the expression of HBD-2 in the gastric corpus of *Hp* carriers compared with *Hp* negative patients. Furthermore, they demonstrated that HBD-1 was expressed in a uniformly low manner, providing further evidence for its accordance with the lack of transcription factor regulatory elements for pro-inflammatory signals in the HBD-1 gene [79].

Recently, it has been determined that HBD3 is always expressed, regardless of *Hp* infection. This study demonstrated that like HBD2, HBD3 is frequently expressed in gastric mucosa with *Hp* infection showing gastritis, but not in inflamed mucosa without *Hp* infection [89].

A potential role of HBD1 in *Hp*-induced gastritis has been postulated by Kocsis et al [88]. They found, in chronic active gastritis patients, a higher frequency of GA and AA genotypes of the G-52A SNP and both inducible and constitutive forms of human defensins are involved in the development of *Hp*-induced gastritis, with DEFB1 expression induced by infection of AGS cells with *cag*PAI strain [88].

*DEFB1* is an important antimicrobial peptide in epithelial tissues and functions as a primary and broad-spectrum response by the host innate defense system [88,90,91]. Multiple studies reported that the low expression of *DEFB1*, due to genetic polymorphisms, is associated with the pathogenesis of digestive diseases [92,93]. In contrast, other studies showed no such correlation between *DEFB1* genetic polymorphisms and digestive diseases [94,95].

**Figure 2:**
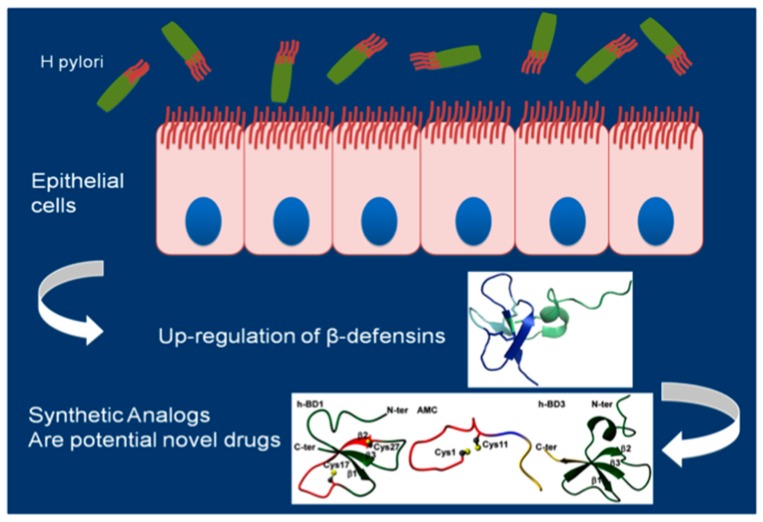
Schematic representation of β-defensins role in *Hp infection. Hp* infection leads to a significant induction of β-defensins that play a pivotal role in the immune response of the gastrointestinal epithelium to *Hp* infection, influencing and activating the adaptive immune system.

HBD4 was expressed at low levels in gastric epithelial cells and was significantly upregulated in infectious and non-infectious gastritis. Standard eradication but not acid suppression therapy significantly decreased HBD4 expression. Cytotoxin-associated gene (*cag*) A positive *Hp* significantly increased the expression of HBD4 whereas *cag*A negative organisms, non-viable bacteria or culture supernatants had no significant effect. Overexpression and downregulation of TLRs was not associated with an altered HBD4 expression. However, blocking experiments revealed an essential role for the p38 mitogen-activated protein kinase [66]. These studies demonstrated that *Hp* induces a differential expression of β-defensins, which are essential effectors of the innate immune response with functional relevance in host defense. These antimicrobial proteins may be less active against *Hp* than against other microorganisms, resulting in a modification of the gastric microbiota composition during host infection. The ability of *Hp* to alter the gastric microbiota of mice is likely determined by several factors, including the genetic background of the mouse, the strain of *Hp*, and the length of infection (Figure 2) [96].

### 3.2. Peptic Ulcer

Epidemiological studies revealed a very strong association between *Hp* infection and duodenal and gastric ulcers. Peptic ulcers are breaks in the lining of the duodenal or gastric mucosa, most commonly caused by *Hp* and nonsteroidal anti-inflammatory drugs. Peptic ulcer disease is associated with significant mortality and complications include hemorrhage and perforation.

Patients with either gastric or duodenal ulcers have less HBD1 and more HBD2 expression than those with completely normal stomach. This pattern is the same as that noted in subjects with gastritis. The marked increase in HBD2 coupled with the decrease in HBD1 expression, was particularly noted in subjects infected with *Hp*. The authors suggest that the increased expression of HBD2 detected in their study probably represents a defensive response by the gastric epithelium aimed at limiting the infection [97].

### 3.3. Gastric Cancer

Gastric cancer is ranked the fifth most common malignancy worldwide, with an estimated 100,000 new cases per year [98]. It can be divided into two subtypes depending on the location: cardia (gastroesophageal junction) and noncardia (the distal stomach). Noncardia gastric cancer is strongly associated with *Hp*, and it is thought that up to 89% may be attributed to *Hp* infection of the normal gastric mucosa. The infection may progress through a series of histologic steps starting from a state of chronic gastritis to atrophic gastritis, intestinal metaplasia, dysplasia, and finally adenocarcinoma [99]. *Hp* and the associated changes in the stomach alter the ecological niche are inhabited by the gastric microbiota. However, the gastric microbiota also competes, as observed in rodents and older people, with *Hp* for a gastric niche, and may play an important role in the progression of disease. More studies involving the microbiota-host-environment interactions are needed to fully understand the role of gastric bacteria in human health and disease.

In addition to their antimicrobial activity, the expression of β-defensins in various tissues and cell types has been linked with chemotaxis and innate immune signaling, adaptive immunity, wound healing, and carcinogenesis. Moreover, there are some evidences of an involvement of defensins in gastric cancer. Recently, induction of HBD-2 and HBD3 mRNA expression by *Hp* has been shown in human gastric adenocarcinoma cell lines (MKN45, APS, AGS, MKN7 MKN45a) [76,77,78,89]. HBD-2 protein was detected in gastric cancers and paired adjacent non-neoplastic tissue showing gastritis from *Hp*-positive patients, but not in specimens from two of *Hp*-negative patients [100].

*Hp*-infected patients express HBD1 at lower levels in the gastric mucosa than the healthy counterparts, but notably, this correlates with an increased burden of infection and a higher inflammatory score. Moreover, the downregulation of HBD1, resulting from an interference in the NF-κB signaling pathway, requires the engagement by the Type IV secretion system of a 5b1 integrin as well as NOD activation in gastric epithelial cells. These mechanisms, with dysregulated production of defensins by host, may ultimately lead to cell modulation and cancerogenesis. Innate immune activation of different cell types by *Hp* is crucial for host defense and might, on the other hand, provide factors promoting DNA damage and cancer [65].

## 4. Antimicrobial Activity of β-Defensins against Hp

Defensin peptides have also been shown to inhibit *Hp* [78,100]. Recent studies have demonstrated that HBD-2 and HBD-3 are potent against *Hp* [90,101]. Nuding et al., tested the susceptibility of three *Hp* DSM strains and one clinical isolate towards the most important antimicrobial peptides of mucosal surfaces [101]. The constitutive defensin HBD1 and inducible defensins HBD2 and HBD4 showed a strong antibacterial effect towards *E. coli* but no or only a marginal impact on *Hp*. Binding experiments showed that HBD2 accumulated on the cell surface of all four *Hp* strains tested even though it seemed to exert no major negative effects on the bacterium’s viability. The binding of HBD2 to the bacteria resulted in limited structural changes but no effective killing and only strain specific, minor growth inhibition of *Hp*. Possibly, as recently shown by Cullen et al. the modification of lipid A by removing phosphate groups, which results in a comparably less negative surface charge, increases the resistance of *Hp* to HBD2 [102].

Interestingly, although ineffective against *Hp*, HBD4 is known to be effective against other bacterial species such as *Enterococcus faecalis* [103]; this perhaps suggests that evolution can contribute to the ability of some bacteria to evade some AMPs. Despite the sustained potency of natural AMPs against bacteria, some bacterial pathogens have also developed resistance to many of these AMPs [104,105,106]. A major barrier to implementing the use of natural AMPs to treat *Hp* infection is the fact that many of the AMPs undergo proteolytic cleavage by both the host digestive components as well as by bacterial enzymes [107]. Despite the fact that resistance to AMPs has been observed, because these molecules have successfully been a key component in combating bacterial infection for millions of years, investigators have come to realize that natural AMP structures may serve as the basis for designing new synthetic AMPs that may overcome some of the challenges seen with natural AMPs.

## 5. Synthetic Amps Analogs

Several synthetic mimetics of natural AMPs have been developed for use as antimicrobials [108,109,110,111]. It is of interest to investigate whether analogs of β-defensins can be generated to obtain more useful antibiotics. In fact, the practical therapeutic use of human defensins is impaired, however, by their size and complexity of disulfide pairing. During the last 10 years, we and others made many efforts and a number of small defensin analogs have been developed demonstrating that they are able to eradicate few pathogens in vitro [112,113,114]; these molecules possess a broad antimicrobial activity although their small size, this characteristic make these analogs potential candidates to develop novel effective and low-cost drugs. We recently evidenced that β-defensins share a common small structural scaffold, namely the γ-core, itself possessing basal antimicrobial activity [115]. This novel finding allows designing smaller and smaller analogs of β-defensins starting from their core.

A class of copolymer compounds referred to as oligo-acyl-lysyl (OAK) peptides mimics the primary structure and function of natural AMPs, but does not form stable secondary structures [109,116,117,118]. OAKs consist of tandem repeats of alternating acyl chains and lysine residues and display a high potential against *Hp* in vitro and in vivo [110,111].

Leszczyńska et al., assessed in vitro the anti-*Hp* potential of WLBU2, a synthetic analog of cathelicidin LL-37 peptide, and the non-peptide antibacterial agent ceragenin CSA-13. They found that CSA-13, but not LL-37 or WLBU2, retained antibacterial activity which was also more resistant to inhibition by isolated host gastric mucins. CSA-13 has a smaller net charge and a unique distribution of this charge over a steroid scaffold and its antibacterial activity of CSA-13 derived by its ability to compromise bacterial membrane integrity [119].

Very recently, another group evaluate the potential anti-*Hp*. *Hp* activity of the synthetic antimicrobial peptide pexiganan, which is an analog of the peptide magainin, and its nanoparticles (PNPs) create in their laboratory. Pexigan is a magainin AMP analog isolated from the skin of the African clawed frog that have broad-spectrum antibacterial activity in vitro. PNPs are particulate mucosal membrane drug delivery systems that penetrate the mucosal membrane in close proximity to the infection site of *Hp*. This study demonstrated that PNPs improved peptide stability in the stomach and more effectively eradicated *Hp* from mice stomachs than pexiganan [120].

Many studies have also evaluated the ability of AMPs combined with conventional ABs to enhance performance, perhaps via increased permeability of the plasma membrane to a conventional AB [121]. The combinatorial use of various antibiotics (tigecycline, moxifloxacin, piperacillin-tazobactam, or meropene), HBD-3 and LL-37 act in a synergistic manner to kill *C. difficile* strains in vitro [122]. Very recently it has been demonstrated that Tilapia Piscidin 4 (TP4), a novel antimicrobial peptide, acts in a synergistic manner with conventional antibiotics against *Hp* [123]. In this study, the authors reported that TP4 has a significant synergistic effect, reducing the MIC of amoxicillin by one-fourth and the MIC of metronidazole and clarithromycin by one-half. The mechanism of TP4 action may be due to strong affinity of TP4 with the negatively-charged molecular structures of the membrane and inducing membrane micellization, loss of membrane integrity accompanied by marked membrane depolarization. Thus, AMPs may be useful in combination therapies to treat *Hp* antibiotic-resistant strains. A better understanding of the mechanisms regarding *Hp* selective antimicrobial resistance and susceptibility against different peptides might help to identify potential targets for novel eradication therapeutics.

## 6. Conclusions

*Hp* is a Gram-negative, spiral-shaped flagellate bacterium that colonizes approximately half of the world’s population. The most common and serious complications of *Hp* infection include peptic ulcer disease (10%), gastric adenocarcinoma (1%–3%), and primary gastric mucosae associated lymphoide tisse lymphoma. Although antimicrobial peptides protect the mucus and mucosa from bacteria, *Hp* is able to colonize the gastric mucus. The human stomach is protected against microbes by a low gastric pH and by the epithelial secretion of antimicrobial peptides as HBDs. HBDs play a pivotal role in the immune response of the gastrointestinal epithelium to *Hp*-infection, directly influencing and activating the adaptive immune system being modulators in infections, as *Hp*. *Hp* infection leads to a significant induction of HBD2, HBD3, and HBD4 while HBD-1, the constitutive expressed β-defensins, is much less pronounced. Several synthetic mimetics of natural AMPs have been developed for use as antimicrobials and a number of small defensin analogs have been developed demonstrating that they are able to eradicate few pathogens in vitro. There is also a class of copolymer compounds referred to as oligo-acyl-lysyl (OAK) peptides that mimics the primary structure and function of natural AMPs but does not form stable secondary structures. They consist of tandem repeats of alternating acyl chains and lysine residues, a novel design, and are effective against *Hp* in vitro.

In conclusion, *Hp* has developed resistance mechanisms against constitutive antimicrobial host factors, such as HBD1, and even factors induced by *Hp* such as HBD2, 3, 4. It may be speculated that analogs of HBDs and more generally of AMPs could be employed in fight against *Hp* infections. *Hp* antibiotic resistance and susceptibility against different analogs might facilitate the identification and selection of potential targets for novel therapeutics.
